# Supplementary material for: A Phenylfurocoumarin Derivative Reverses ABCG2-Mediated Multidrug Resistance In Vitro and In Vivo
Source: Int J Mol Sci. 2021 Nov 19;22(22):12502. doi: 10.3390/ijms222212502 (PMC8618058; doi:10.3390/ijms222212502)
Supplement: Supplementary file 1 [file ijms-22-12502-s001.zip › ijms-1408018-supplementary.pdf]

Table S1. The effect of candidate compounds on the reversal of ABCB2-mediated resistance to SN38 in HCT-116/BCRP cells

	IC_{50} ± SE (μM)[a]	fold reversal[b]		IC_{50} ± SE (μM)[a]	fold reversal[b]
Medium	2.59 ± 0.24	1			
Ko143	0.26 ± 0.027	10.1			
No.01 5uM	2.36 ± 0.21	1.1	No.01 10uM	1.34 ± 0.14	1.9
No.03 5uM	2.88 ± 0.26	0.9	No.03 10uM	2.14 ± 0.24	1.2
No.04 5uM	2.67 ± 0.10	1.0	No.04 10uM	2.02 ± 0.18	1.3
No.06 5uM	3.50 ± 0.23	0.7	No.06 10uM	2.66 ± 0.45	1.0
No.07 5uM	0.61 ± 0.12	4.3	No.07 10uM	0.22 ± 0.036	11.6
No.08 5uM	1.69 ± 0.16	1.5	No.08 10uM	1.17 ± 0.16	2.2
No.09 5uM	2.70 ± 0.39	1.0	No.09 10uM	1.14 ± 0.023	2.3
No.14 5uM	1.05 ± 0.16	2.5	No.14 10uM	0.63 ± 0.068	4.1
No.15 5uM	1.31 ± 0.35	2.0	No.15 10uM	0.62 ± 0.11	4.2
No.16 5uM	0.91 ± 0.12	2.9	No.16 10uM	0.56 ± 0.072	4.6
No.17 5uM	0.93 ± 0.15	2.8	No.17 10uM	0.86 ± 0.12	3.0
No.20 5uM	0.73 ± 0.16	3.5	No.20 10uM	0.67 ± 0.10	3.9
**No.22 5uM**	**0.36 ± 0.11**	**7.3**	**No.22 10uM**	**0.19 ± 0.018**	**13.8**
No.23 5uM	1.30 ± 0.17	2.0	No.23 10uM	0.87 ± 0.10	3.0

[a]The values are mean ± SE of three independent experiments performed in triplicate. [b]The fold reversal of MDR was calculated by dividing the IC_{50} for cells with the SN-38 (Medium) in the absence of the compound (ABCG2 inhibitor) by that obtained in the presence of the compound. No.22 is a phenylfurocoumarin derivative. No.11 and No.18 were cytotoxic, therefore IC_{50} could not be calculated (data not shown).

Table S2. Cytotoxicity of Ko143 and oxypeucedanin

	IC_{50} ± SE (μM)[a] HCT-116/BCRP	fold reversal[b]
Medium	2.27 ± 0.093	1.0
Ko143 1 μM	0.17 ± 0.012	13.2
oxypeucedanin 5 μM	0.97 ± 0.014	2.3
oxypeucedanin 10 μM	0.76 ± 0.049	3.0

[a]Values are mean ± SE. [b]The fold reversal of MDR was calculated by dividing the IC_{50} for cells with the SN-38 (Medium) in the absence of the reversing agent by that obtained in the presence of that agent.
